# Supplementary material for: Epigenetic divergence during early stages of speciation in an African crater lake cichlid fish
Source: Nat Ecol Evol. 2022 Oct 20;6(12):1940–51. doi: 10.1038/s41559-022-01894-w (PMC9715432; doi:10.1038/s41559-022-01894-w)
Supplement: Supplementary file 1 — Supplementary Notes and Tables. [file 41559_2022_1894_MOESM1_ESM.pdf]

nature ecology & evolution

Article

https://doi.org/10.1038/s41559-022-01894-w

Epigenetic divergence during early stages of speciation in an African crater lake cichlid fish

In the format provided by the authors and unedited

SUPPLEMENTARY INFORMATION

Epigenetic Divergence during Early Stages of Speciation in an African Crater Lake Cichlid Fish

Grégoire Vernaz[1,2,3] *, Alan G. Hudson[4,8], M. Emília Santos[5], Bettina Fischer[2], Madeleine Carruthers[4], Asilatu H. Shechonge[6], Nestory P. Gabagambi[6], Alexandra M. Tyers[7,9], Benjamin P. Ngatunga[6], Milan Malinsky[3,10], Richard Durbin[2,3], George F. Turner[7], Martin J. Genner[4,11]* & Eric A. Miska[1,2,3,11]*

[1]Wellcome/CRUK Gurdon Institute, University of Cambridge, Cambridge CB2 1QN, UK;
[2]Department of Genetics, University of Cambridge, Cambridge CB2 3EH, UK;
[3]Wellcome Sanger Institute, Hinxton CB10 1SA, UK;
[4]School of Biological Sciences, University of Bristol, Bristol BS8 1TQ, UK;
[5]Department of Zoology, University of Cambridge, Cambridge CB2 3EJ, UK;
[6]Tanzania Fisheries Research Institute (TAFIRI), Dar es Salaam, Tanzania;
[7]School of Natural Sciences, Bangor University, Bangor Gwynedd LL57 2UW, UK;
[8]Present address: School of Life Sciences, University of Hawai'i at Mānoa, Honolulu, USA;
[9]Present address: Max Planck Institute for Biology of Ageing, Cologne 50931, Germany;
[10]Present address: Institute of Ecology and Evolution, University of Bern, 3012 Bern, Switzerland.
[11]Equal contribution

*Correspondence: **M.J.G** (m.genner[at]bristol.ac.uk), **E.A.M.** (eam29[at]cam.ac.uk), **G.V** (gv268[at]cam.ac.uk)

ORCID

**GV:** 0000-0001-8942-2370	**MM:** 0000-0002-1462-6317
**AGH:** 0000-0001-6436-2025	**RD:** 0000-0002-9130-1006
**MES:** 0000-0003-3158-7935	**GFT:** 0000-0003-0099-7261
**AS**: 0000-0002-9228-0323	**MJG:** 0000-0003-1117-9168
**NPG:** 0000-0003-0260-2972	**EAM:** 0000-0002-4450-576X

Table of Contents

SUPPLEMENTARY NOTES 2
Isotope analysis 2
RRBS-WGBS cross-validation 2
SUPPLEMENTARY TABLES 3

Isotope analysis

We statistically confirmed that the δ^{13}C for each of the three populations followed a normal/Gaussian distribution by performing Shapiro-Wilk test (Benthic [B]: W= 0.95, p=0.7; Shallow [S]: W= 0.96, p=0.73; Riverine [R]: W=0.93, p=0.51). We then showed the δ^{13}C of populations had unequal variance (heteroscedasticity) by performing Levene's test for homogeneity of variance ($F_{2, 30}$=3.7, p=0.036). As the samples for each population did not statistically deviate from normal Gaussian distributions, we used the parametric one-way ANOVA (Welch's ANOVA test: $F_{2, 14.83}$= 111.56, two-sided p= 1.154e-09) to test for statistically significant mean differences between the three groups (small group size, Gaussian distribution and heterogenous variance), followed by post hoc pairwise comparisons of populations using Games-Howell multiple tests with p-value adjustment using Turkey's method, robust for small group sizes (all pairwise comparisons, two-sided p<0.008; see Fig.1d). Finally, we computed the 95% Confident Interval (CI) of unpaired mean difference between each pairwise group comparison to further provide descriptive statistics using DABEST[1] (5,000 bootstrap resamples): mean differences [95%IC mean difference]: River vs Littoral: -2.24 [95CI -3.43; -1.06] ; River vs Benthic: -6.62 [95CI -7.62; -5.74]; Littoral vs Benthic: -4.38 [95CI -5.25; -3.58]. Altogether, this robustly indicates that the three populations have statistically different δ^{13}C isotope profiles, in line with different food sources/diets (Fig.1d). See Methods for details on statistical computer programmes used.

RRBS-WGBS cross-validation

To cross-compare RRBS and WGBS datasets and validate the use of whole-genome unbiased methylome sequencing technique, methylation variation at WGBS DMRs (n=413 DMRs in total) within the RRBS data was assessed. The limited coverage overlap of WGBS-DMRs by RRBS datasets is primarily explained by the reduced representation of the genome that RRBS technique provides. Nevertheless, these data enabled us to show that methylome variation at WGBS-DMRs using RRBS samples is highly population-specific (Extended Data Fig. 4a), in line with the genome-wide (WGBS) methylome variation (Fig. 1f). We were also able to show that methylation levels at all DMRs found between pairwise WGBS groups (from Fig.1g) were highly correlated between RRBS and WGBS sample groups (averaged mCG/CG over WGBS-DMRs for both RRBS and WGBS samples; Extended Data Fig. 4b), providing evidence that WGBS methylome divergence is recapitulated at the population-levels using RRBS samples.

Furthermore, in total, using the RRBS samples only, we identified 333, 534 and 342 DMRs between River-Littoral, River-Benthic and Littoral-Benthic fish, respectively (Extended Data Figure 4d). On average, ~4.4-23.1% of those RRBS-DMRs directly overlapped with WGBS-DMRs (Extended Data Figure 4d). The lower number of RRBS-DMRs compared to WGBS-DMRs, as well as the limited overlap, is likely to primarily stem from the difference in sample size.

As WGBS data enables unbiased, genome-wide assessment of methylome divergence, and since it was highly representative of methylome variation observed at a population level (n=11-12 RRBS independent biological replicates per group), we chose to use the WGBS dataset for all downstream experiments.

References

1. Ho, J., Tumkaya, T., Aryal, S., Choi, H. & Claridge-Chang, A. Moving beyond P values: data analysis with estimation graphics. *Nat. Methods* **16**, 565–566 (2019).

Supplementary Table 1. Sequencing summary for RRBS dataset, including total sequencing read count and mapping rates

See excel workbook

Supplementary Table 2. Sequencing summary for WGBS dataset, including total sequencing read count and mapping rates

See excel workbook

Supplementary Table 3. Sequencing summary for RNAseq dataset, including total sequencing read count and mapping rates

See excel workbook

Supplementary Table 4. Isotope measurements ($\delta^{13}C$) for the three populations.

See excel workbook
