# Supplementary material for: Epigenetic divergence during early stages of speciation in an African crater lake cichlid fish
Source: Nat Ecol Evol. 2022 Oct 20;6(12):1940–51. doi: 10.1038/s41559-022-01894-w (PMC9715432; doi:10.1038/s41559-022-01894-w)
Supplement: Supplementary file 2 — Reporting Summary [file 41559_2022_1894_MOESM2_ESM.pdf]

nature portfolio

Corresponding author(s): EAM, GV, MJG

Last updated by author(s): Jul 20, 2022

Reporting Summary

Nature Portfolio wishes to improve the reproducibility of the work that we publish. This form provides structure for consistency and transparency in reporting. For further information on Nature Portfolio policies, see our Editorial Policies and the Editorial Policy Checklist.

Statistics

For all statistical analyses, confirm that the following items are present in the figure legend, table legend, main text, or Methods section.

n/a	Confirmed	
☐	☒	The exact sample size (*n*) for each experimental group/condition, given as a discrete number and unit of measurement
☐	☒	A statement on whether measurements were taken from distinct samples or whether the same sample was measured repeatedly
☐	☒	The statistical test(s) used AND whether they are one- or two-sided<br>*Only common tests should be described solely by name; describe more complex techniques in the Methods section.*
☐	☒	A description of all covariates tested
☐	☒	A description of any assumptions or corrections, such as tests of normality and adjustment for multiple comparisons
☐	☒	A full description of the statistical parameters including central tendency (e.g. means) or other basic estimates (e.g. regression coefficient) AND variation (e.g. standard deviation) or associated estimates of uncertainty (e.g. confidence intervals)
☐	☒	For null hypothesis testing, the test statistic (e.g. *F*, *t*, *r*) with confidence intervals, effect sizes, degrees of freedom and *P* value noted<br>*Give P values as exact values whenever suitable.*
☒	☐	For Bayesian analysis, information on the choice of priors and Markov chain Monte Carlo settings
☒	☐	For hierarchical and complex designs, identification of the appropriate level for tests and full reporting of outcomes
☐	☒	Estimates of effect sizes (e.g. Cohen's *d*, Pearson's *r*), indicating how they were calculated

Our web collection on statistics for biologists contains articles on many of the points above.

Software and code

Policy information about availability of computer code

Data collection: No software was used to collect data.

Data analysis: TrimGalore v.0.6.2, bismark v.0.20.0, kallisto v0.46.0, bedtools v2.27.1, R v.3.6.3 (R packages: DSS v.2.32.0, pheatmap v1.0.12, ggplot2 v.3.3.0, tidyverse v1.3.1, FSA v0.8.25, sleuth v0.30.0,deepTools v3.2.1, phangorn v2.5.5, circlize v0.4.12, rstatix v0.7.0), IGV v2.9.2, RepeatMasker v4.0.9.p2, makeCGI, g:Profiler (e100_eg47_p14_7733820), homer-4.9.1-6, bedGraphToBigWig (v4), lastz v1.02, UCSC liftOver tool (which included scripts: axtChain and liftOver; kent source version 418)

For manuscripts utilizing custom algorithms or software that are central to the research but not yet described in published literature, software must be made available to editors and reviewers. We strongly encourage code deposition in a community repository (e.g. GitHub). See the Nature Portfolio guidelines for submitting code & software for further information.

Data

Policy information about availability of data

All manuscripts must include a data availability statement. This statement should provide the following information, where applicable:

- Accession codes, unique identifiers, or web links for publicly available datasets
- A description of any restrictions on data availability
- For clinical datasets or third party data, please ensure that the statement adheres to our policy

WGBS, RRBS and RNAseq raw sequencing data have been deposited in the Gene Expression Omnibus (GEO) database under the accession number GSE174120 . Refer to Supplementary Tables 1-3 for sample IDs.

Field-specific reporting

Please select the one below that is the best fit for your research. If you are not sure, read the appropriate sections before making your selection.

☐ Life sciences ☐ Behavioural & social sciences ☒ Ecological, evolutionary & environmental sciences

For a reference copy of the document with all sections, see nature.com/documents/nr-reporting-summary-flat.pdf

Ecological, evolutionary & environmental sciences study design

All studies must disclose on these points even when the disclosure is negative.

Study description	This study aimed at generating high-coverage whole-genome bisulphite sequencing and total RNA sequencing of liver tissues from wild-caught and tank-reared Lake Masoko and River Mbaka A.calliptera cichlid specimens to investigate epigenetic divergence and inheritance during early stages of speciation.
Research sample	In total, wild-caught A. calliptera specimens from Lake Masoko (both littoral/yellow and benthic/blue ecomorph populations) and from the neighboring river Mbaka (related to the ancestral population of Lake Masoko ecomorphs) were used in this study. In addition, tank-reared specimens from the three A.calliptera populations were used as well (first generation, bred from wild caught parental lines). Only adult males in full nuptial coloration were used in this study. DNA and RNA samples were extracted from liver tissues.<br><br>For RRBS experiments, 11-12 wild caught specimens for each of the three A. calliptera populations were used. For WGBS, 2-3 wild/tank-reared specimens were used for each of the three populations (wild WGBS samples were the same as for RRBS dataset). For RNAseq, 4-5 wild specimens were used for each population. Only males specimens were used.
Sampling strategy	The main strategy was to select 2-3/11-12/4-5 (WGBS/RRBS/RNAseq) adult, males (independent biological replicates) for each of the three A.calliptera populations (littoral and benthic populations from Lake Masoko and the riverine population of River Mbaka) in order to assess methylome and transcriptome divergence during early stages of speciation in this Crater Lake cichlid system. No statistical method was used to define sample size - sample sizes were based on literature. Within-species variation for all analyses and statistical procedures was taken into account. All fish were size-matched wild caught male specimens displaying full nuptial colorations (when males) and collected by collaborators.
Data collection	Fish were identified (photographs), registered in an excel sheet and dissected by AMT, GV, AGH, MC and MES. DNA extractions were performed by GV, AGH and MC. RNA extractions were performed by GV and BF. Illumina HiSeq sequencing was performed by the sequencing facility at CRUK/CI, Cambridge UK (WGBS), by the sequencing facility of the Wellcome Sanger Institute (RNAseq) and the sequencing facility of the University of Bristol (RRBS). All sequencing data was analyzed by GV.
Timing and spatial scale	Wild specimens were caught by professional divers in 2015, 2016, 2018 and 2019 in Tanzania in collaboration and compliance with the Tanzania Fisheries Research Institute (various collaborative projects). Upon collection, tissues were immediately dissected and placed in RNAlater (Sigma), and were then stored at -80°C upon return. Tank-reared specimens were culled at the same time according to the ethical and veterinary regulations in place at the University of Bangor, using MS222 method in 2017-2018.
Data exclusions	No data were excluded fro the analysis.
Reproducibility	All attempts to reproduce the experiments were successful.
Randomization	Species were selected based on their respective ecological niches and morphologies (using published literature). Randomization was not appropriate.
Blinding	All analyses related to genome-wide methylome and transcriptome differences (including hierarchical clustering, principal component analysis) were performed in an unbiased, blind approach (populations not known). For other analysis (DMR, DEG, isotope), investigators were aware of the population identity.

Did the study involve field work? ☐ Yes ☒ No

Reporting for specific materials, systems and methods

We require information from authors about some types of materials, experimental systems and methods used in many studies. Here, indicate whether each material, system or method listed is relevant to your study. If you are not sure if a list item applies to your research, read the appropriate section before selecting a response.

Materials & experimental systems

n/a	Involved in the study
☒	☐ Antibodies
☒	☐ Eukaryotic cell lines
☒	☐ Palaeontology and archaeology
☐	☒ Animals and other organisms
☒	☐ Human research participants
☒	☐ Clinical data
☒	☐ Dual use research of concern

Methods

n/a	Involved in the study
☒	☐ ChIP-seq
☒	☐ Flow cytometry
☒	☐ MRI-based neuroimaging

Animals and other organisms

Policy information about studies involving animals; ARRIVE guidelines recommended for reporting animal research

Laboratory animals	Tank-reared A. calliptera (river, benthic and littoral morphs), first generation (G1) were bred from wild specimens. Only adult males displaying full nuptial coloration were used for all experiments (two males per population).
Wild animals	All wild specimens of the three A.calliptera populations (benthic, littoral, river) were caught by professional SCUBA divers using fixed gill nets (at specific depths) in compliance permits issued to GF Turner, MJ Genner R Durbin, EA Miska by the Tanzania Fisheries Research Institute.
Field-collected samples	Wild specimens of A.calliptera (benthic, littoral and river) were imported to the UK by aquarium-trade import specialists. Specimens were cared for, reared and bred in the fish facilities of the University of Bangor in compliance with the ethical and veterinary regulations in place. Fish were reared under the same controlled laboratory conditions in separate tanks (light/dark 12/12 cycles, diet: algae flakes daily, 2-3times weekly frozen diet). Only adult males (2 per population) displaying full nuptial colouration were culled (MS222 anesthesia) for this study.
Ethics oversight	Sampling collection and shipping were approved by permits issued to GF Turner, MJ Genner R Durbin, EA Miska by the Tanzania Fisheries Research Institute. Tank-reared fish populations were maintained in compliance with the ethical and veterinary regulations in place at the University of Bangor.

Note that full information on the approval of the study protocol must also be provided in the manuscript.
